# Relationship between FEV_1 _change and patient-reported outcomes in randomised trials of inhaled bronchodilators for stable COPD: a systematic review

**DOI:** 10.1186/1465-9921-12-40

**Published:** 2011-04-08

**Authors:** Marie Westwood, Jean Bourbeau, Paul W Jones, Annamaria Cerulli, Gorana Capkun-Niggli, Gill Worthy

**Affiliations:** 1 Kleijnen Systematic Reviews Ltd., York, UK; 2 Respiratory Epidemiology and Clinical Research Unit, McGill University, Montreal, Canada; 3 St George's University Medical School, University of London, UK; 4 Novartis Pharma AG, Basel, Switzerland

## Abstract

**Background:**

Interactions between spirometry and patient-reported outcomes in COPD are not well understood. This systematic review and study-level analysis investigated the relationship between changes in FEV_1 _and changes in health status with bronchodilator therapy.

**Methods:**

Six databases (to October 2009) were searched to identify studies with long-acting bronchodilator therapy reporting FEV_1 _and health status, dyspnoea or exacerbations. Mean and standard deviations of treatment effects were extracted for each arm of each study. Relationships between changes in trough FEV_1 _and outcomes were assessed using correlations and random-effects regression modelling. The primary outcome was St George's Respiratory Questionnaire (SGRQ) total score.

**Results:**

Thirty-six studies (≥3 months) were included. Twenty-two studies (23,654 patients) with 49 treatment arms each contributing one data point provided SGRQ data. Change in trough FEV_1 _and change in SGRQ total score were negatively correlated (r = -0.46, p < 0.001); greater increases in FEV_1 _were associated with greater reductions (improvements) in SGRQ. The correlation strengthened with increasing study duration from 3 to 12 months. Regression modelling indicated that 100 mL increase in FEV_1 _(change at which patients are more likely to report improvement) was associated with a statistically significant reduction in SGRQ of 2.5 (95% CI 1.9, 3.1), while a clinically relevant SGRQ change (4.0) was associated with 160.6 (95% CI 129.0, 211.6) mL increase in FEV_1_. The association between change in FEV_1 _and other patient-reported outcomes was generally weak.

**Conclusions:**

Our analyses indicate, at a study level, that improvement in mean trough FEV_1 _is associated with proportional improvements in health status.

## Introduction

Chronic obstructive pulmonary disease (COPD) is a complex, chronic condition, which is characterised by progressive airflow limitation that is not fully reversible. The major symptoms of COPD, such as dyspnoea, cough and sputum production, are disabling and have substantial impact on both patients' health status and the health care system [1,2]. Although treatment involves several approaches, bronchodilator medications are central to the management of COPD, improving both lung function and symptoms [1].

The complex nature of COPD means that it is important to assess treatment effectiveness in terms of patient-reported outcomes, including symptoms or health status scores [3]. Clinicians and policy makers have recognised the importance of measuring health status, in order to make informed patient management and policy decisions [4], and clinician-led guidelines recommend this approach for COPD [1,2]. However, regulatory authorities continue to emphasise airflow obstruction, measured by spirometry, as the primary outcome required for registration trials of new bronchodilators. It is therefore relevant to establish if and how changes in lung function may translate into patient-reported outcomes.

Although primary studies with bronchodilators frequently report both spirometry and patient-reported outcomes, the relationships between outcome measures are poorly understood. A study by Stahl et al. published in 2001, showed weak correlations between the St George's Respiratory Questionnaire (SGRQ) and cough, breathlessness, forced expiratory volume in 1 second (FEV_1_) and walking distance but reported only limited supporting patient level data [5]. Study-level meta-analysis is a meaningful and cost-effective approach to addressing a clinical research question, particularly where individual patient data is difficult to obtain [6]. We are unaware of any study level analysis which has specifically addressed how lung function is related to outcomes.

The present study was a systematic review of randomised controlled trials (RCTs) of inhaled bronchodilators in adult patients with stable COPD, which reported change in trough FEV_1_, the primary physiological outcome in most studies of long-acting bronchodilators, alongside patient-reported outcomes. The primary objective was to assess at a study level the relationship between FEV_1 _change and health status change, as measured by the SGRQ, and to estimate the increase in mean FEV_1 _associated with a clinically important improvement in health status. As secondary objectives, we assessed the relationship between change in FEV_1 _and SGRQ domains, the influence of study duration, and the relationship between change in FEV_1 _and change in other patient-reported outcomes, such as dyspnoea, as measured by the Transition Dyspnea Index (TDI), and COPD exacerbations.

## Methods

### Search strategy

We sought all relevant trials regardless of language or publication status (published, unpublished, in press, and in progress). The following databases were searched: MEDLINE (1980 to March 2009); EMBASE (1980 to March 2009); "Cochrane Reviews" (CDSR, Cochrane Library issue 4 2009); "Clinical Trials" (CENTRAL, Cochrane Library issue 4 2009); DARE (March 2009, CRD website); and HTA (March 2009, CRD website). Search strategies with keywords were developed specifically for each database: the search strategy for MEDLINE is provided in Additional file 1. In addition, databases of completed and ongoing trials such as ClinicalTrials.gov, websites of licensing agencies, the Guidelines International Network and worldwide HTA were searched and references in retrieved articles and systematic reviews were checked.

### Selection criteria

Our selection criteria included published and unpublished, parallel, RCTs of ≥12 weeks duration. Non-RCTs were excluded, given that RCTs represent the most robust level of efficacy evidence, especially for outcomes reported by patients. Studies had to include COPD patients (according to any definition) aged ≥35 years with stable disease (no exacerbations for at least 4 weeks prior to study entry or 'stable COPD' as an inclusion criteria), chronic bronchitis (excluding acute/spastic bronchitis), or emphysema. Trials which recruited mixed populations (e.g. asthma and COPD) were excluded, unless separate data were reported for COPD patients.

We included all studies that had intervention treatment arms using a long-acting inhaled bronchodilator treatment as monotherapy for stable COPD, e.g. long-acting β2-agonists (LABA), long-acting muscarinic antagonists (LAMA), LABA + LAMA combinations, methylxanthines and placebo, thus limiting the analysis to drugs with similar pharmacodynamic properties. The comparator treatment could include a placebo or any of the interventions listed above. Short-acting treatment arms were excluded. Studies had to report change in trough FEV_1 _from baseline and at least one patient-reported outcome (health status [SGRQ], exacerbations or dyspnoea [TDI]). Trough FEV_1 _was extracted as reported in the primary studies. Although there was some variation in details provided, this was usually defined as the measurement of FEV_1 _taken before the first morning dose. Both the SGRQ and TDI are disease specific questionnaires. The SGRQ consists of three domains (Symptoms, Activity and Impacts) and a Total score which provides values between 0 and 100. Higher values correspond to greater impairment, with a 4 unit change in total score considered to be the minimal clinically important difference (MCID) [7]. The TDI represents a change from baseline and provides values between -9 and 9 with positive values indicating improvement and a 1 unit change representing the MCID [8].

### Trial selection, data extraction and quality assessment

Two reviewers (MW and GW) independently inspected the abstract of each reference identified to determine potential relevance. For potentially relevant articles, or in cases of disagreement, the full article was obtained, independently inspected, and inclusion criteria applied. Any disagreement was resolved through discussion and checked by a third reviewer. Data for each study were extracted by one reviewer and checked for accuracy by a second reviewer, using a standardised data extraction sheet. Any disagreements were resolved by consensus. Baseline and endpoint data were extracted where available, otherwise, change from baseline data were extracted. Outcome data were extracted for all available time points. If studies did not report numerical data, values were estimated from graphs, and standard deviations were imputed using weighted averages from other studies which included the same drug comparison and time point, in line with recommended methodology [9].

Quality assessment was carried out by one reviewer, using the Cochrane Collaboration quality assessment checklist, and checked for accuracy by a second reviewer. Any disagreements were resolved by consensus. Results are summarised in Additional file 2.

### Data analysis

The relationship between mean changes in FEV_1 _and mean changes in SGRQ scores for each treatment arm from each study was assessed visually using scatter plots. Plots were constructed for SGRQ total score and SGRQ domains (Symptoms, Activity and Impacts) at any time point measured; where studies reported multiple time points, only data for the 6 month time point (the most frequently measured time point across studies) were used for analyses that include all time points. For the relationship between changes in FEV_1 _and SGRQ total score, separate plots were constructed for the 3, 6 and 12 month time points. Pearson correlation coefficients were calculated and regression lines from a simple linear regression model were added to each plot. These were used to estimate the mean change in FEV_1 _corresponding to 3- and 4-unit changes in SGRQ, and the mean change in SGRQ score associated with a 100 mL increase in FEV_1 _(magnitude of change in FEV_1 _at which patients are more likely to report improvement in an important clinical parameter such as health status) [10].

Random effects regression modelling was used to explore the effects of the change in FEV_1 _on the change in total SGRQ score. The model included time (3, 6 or 12 months) as a categorical variable and study was treated as a random effect to allow for correlation within each study, thus adjusting for possible confounders. This model allows an estimate of the strength of the relationship between FEV_1 _and SGRQ (the size and statistical significance of the model coefficient). Where sufficient data were available, similar methods were applied to investigate the relationship between changes in FEV_1 _and the outcomes TDI and percentage of patients experiencing at least one COPD exacerbation. All statistical analyses were performed in Stata 10.1.

## Results

### Overview of included studies

**Figure 1:**
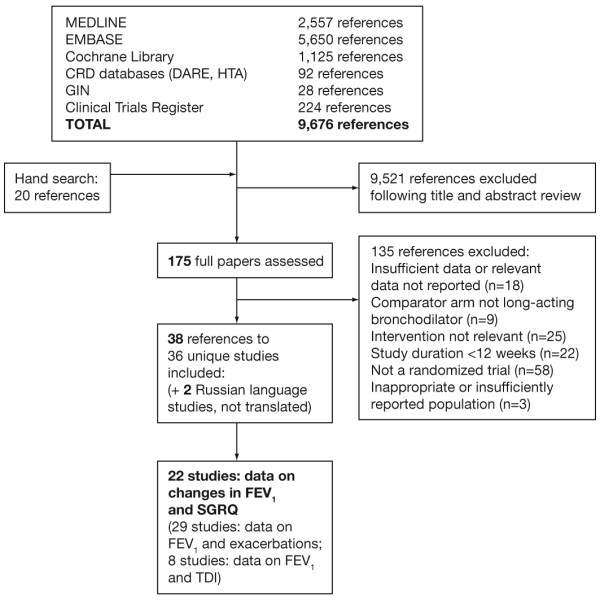
**Flow of studies through the review process**. Abbreviations: MEDLINE, medical literature analysis and retrieval system online; EMBASE, Excerpta Medica database; CRD, Centre for Reviews and Dissemination; DARE, Database of Abstracts of Reviews of Effects; HTA, Health Technology Assessment; GIN, Guidelines International Network.

The search strategy initially yielded 9676 references. Figure 1 illustrates the flow of studies through the review process. After screening for potential relevance, 175 full papers were assessed for possible inclusion. From these, 36 studies met the inclusion criteria [5,11-45]. A further two references were identified to be duplicates of previously identified studies [46,47]. Twenty-two studies with 49 treatment arms contributed to the primary analyses exploring the relationship between changes in FEV_1 _and SGRQ scores [5,11-31]. Twenty nine studies provided data on exacerbations [11-13,16-22,25,26,29-45] and eight studies provided data on dyspnoea [11-13,21,26,30,33,41]. All studies were parallel, RCTs of LAMA (tiotropium) and/or LABA (salmeterol, formoterol, arformoterol) with or without a placebo arm.

Table 1 shows the study characteristics for studies providing data on FEV_1 _and SGRQ scores, exacerbations, or dyspnoea. The 49 data sets for the SGRQ analyses included 23,654 patients, of whom 72% were male with an average age of 64 years, and mean baseline FEV_1 _43% predicted.

**Table 1:** Description of studies providing data on FEV_1 _and SGRQ, dyspnoea (TDI), or exacerbations for long-acting bronchodilators

Study	Duration, (months)*	Number randomised (by treatment)	Age, (years)	Male, (%)	Smoking history, (pack years)	FEV_1 _% predicted	Outcomes reported			
							
							SGRQ total	SGRQ domains	Number with ≥ 1 exacerbation	TDI
Aaron 2007 [11]	12	304 (T 156, T + S 148)	65.9 (8.6)	56	50.3 (27.6)	41.7 (13.3)	Yes	No	Yes	Yes
Baumgartner 2007 [12]	3	428 (A 141, S 144, Pl 143)	62.9 (9.0)	58	NR	40.8 (12.7)	Yes	Yes	Yes	Yes
Beeh 2006 [32]	3	1639 (T 1236, Pl 403)	62.2 (8.7)	76	35.8 (19.5)	45.5 (14.9)	No	No	Yes	No
Boyd 1997 [33]	4	456 (S 229, Pl 227)	61.5	79	NR	NR	No	No	Yes	Yes
Briggs 2005 [34]	3	653 (T 328, S 325)	64.4 (6.3)	67	55.9 (28.8)	37.7 (12.1)	No	No	Yes	No
Brusasco 2003 [13]	6	1207 (T 402, S 405, Pl 400)	64.2 (8.4)	76	43.8 (23.2)	38.5 (11.8)	Yes	No	Yes	Yes
Calverley 2003 [14]	12	733 (S 372, Pl 361)	63.3 (8.6)	72	43.6 (22.2)	44.3 (13.8)	Yes	No	No	No
Calverley 2007 [15]	12	6184 (S 1521, Pl 1524)	65.0 (8.2)	76	49.0 (27.3)	43.9 (12.5)	Yes	No	No	No
Campbell 2005 [16]	6	442 (F 225, Pl 217)	60	68	37	53.8	Yes	No	Yes	No
Casaburi 2000 [35]	3	470 (T 279, Pl 191)	65.2 (8.8)	65	62.9 (32.0)	39.0 (13.9)	No	No	Yes	No
Casaburi 2002 [17]	12	921 (T 550, Pl 371)	65.0 (9.0)	65	61.0 (30.5)	38.6 (13.9)	Yes	Yes	Yes	No
Chan 2007[18]	12	913 (T 608, Pl 305)	66.8 (8.8)	60	50.4 (23.9)	39.4 (13.5)	Yes	Yes	Yes	No
Chapman 2002 [19]	6	408 (S 201, Pl 207)	NR	64	38	45	Yes	Yes	Yes	No
Covelli 2005 [36]	3	196 (T 100, Pl 96)	64.6 (9.0)	58	65.5 (33.4)	39.4 (13.4)	No	No	Yes	No
Dahl 2001 [20]	6	392 (F 192, Pl 200)	63.5 (8.4)	77	41.8	45.0 (12.7)	Yes	Yes	Yes	No
Donohue 2002 [21]	6	623 (T 209, S 213, Pl 201)	64.9 (7.9)	75	47.0 (25.0)	42.3 (9.3)	Yes	Yes	Yes	Yes
Donohue 2008 [37]	12	793 (Af 528, S 265)	64.2 (8.8)	59	NR	38.0 (13.1)	No	No	Yes	No
Dusser 2006 [38]	12	1010 (T 500, Pl 510)	64.8 (9.3)	88	NR	47.9 (12.7)	No	No	Yes	No
Freeman 2007 [39]	3	395 (T 200, Pl 195)	64.9 (9.1)	54	37.4 (17.3)	48.9 (10.6)	No	No	Yes	No
Gross 2008 [22]	3	351 (F 123, Pl 114)	62.7 (8.9)	58	NR	44.5 (12.1)	Yes	Yes	Yes	No
Johansson 2008 [40]	3	224 (T 107, Pl 117)	61.5 (8.3)	48	31.5 (12.1)	73.4 (12.6)	No	No	Yes	No
Jones 1997 [23]	3	189 (S 94, Pl 95)	62.5 (8.0)	79	NR	46.0 (15.0)	Yes	No	No	No
Mahler 1999 [41]	3	411 (S 135, I 133, Pl 143)	63.5 (8.5)	74	60.2 (32.5)	40.0	No	No	Yes	Yes
Moita 2008 [42]	3	311 (T 147, Pl 164)	64.3 (8.6)	95	55.0 (23.6)	41.4 (14.1)	No	No	Yes	No
Niewoehner 2005 [43]	6	1829 (T 914, Pl 915)	67.9 (8.6)	99	68.4 (36.0)	35.6 (12.6)	No	No	Yes	No
Rennard 2009 [24]	12	976 (F 495, Pl 481)	63.0 (9.1)	65	NR	40.1 (11.7)	Yes	Yes	No	No
Rossi 2002 [25]	12	645 (F 214, Pl 220)	62.7	83	NR	47.7	Yes	Yes	Yes	No
Sepracor inc. NCT00250679 2009 [26]	6	296 (F 147, Af 149)	64.7 (8.4)	61	NR	41.0 (12.6)	Yes	No	Yes	Yes
Stahl 2001 [5]	3	121 (F 61, Pl 60)	64	52	NR	33.3	Yes	No	No	No
Stockley 2006 [27]	12	726 (S 316, Pl 318)	62.4 (9.2)	67	39.7 (21.6)	46.0 (14.3)	Yes	Yes	No	No
Tashkin 2008 [28]	6	584 (F 284, Pl 300)	63.4 (9.6)	67	NR	40.4 (12.5)	Yes	Yes	No	No
Tashkin 2008 [29]	12	5993 (T 2987, Pl 3006)	64.5 (8.5)	75	48.7 (28.0)	39.4 (12.0)	Yes	No	Yes	No
Tashkin 2009 [30]	3	255 (T + F 124, T 131)	63.8 (8.6)	66	NR	NR	Yes	Yes	Yes	Yes
Tonnel 2008 [31]	9	554 (T 266, Pl 288)	64.2 (9.9)	86	43.7 (21.9)	46.8 (12.8)	Yes	Yes	Yes	No
van Noord 2000 [44]	3	97 (S 47, Pl 50)	74.0 (6.5)	88	NR	40.3 (10.7)	No	No	Yes	No
Vogelmeier 2008 [45]	6	847 (F 210, T 221, F + T 207, Pl 209)	62.6 (8.8)	78	38.0 (19.7)	51.2 (9.9)	No	No	Yes	No

Data are mean (SD) unless otherwise stated. *time point used in analyses

Af: arformoterol, F: formoterol, FEV_1_: forced expiratory volume in one second, NR: not reported, Pl: placebo, S: salmeterol, SGRQ: St George's Respiratory Questionnaire, T: tiotropium, TDI: Transition Dyspnea Index

### FEV_1 _change and change in SGRQ total score

**Figure 2:**
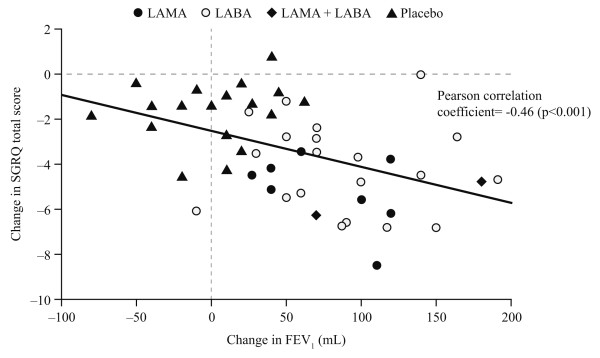
**Scatter plot of mean change in FEV**_**1**_**, for all treatments (LAMA, LABA, LAMA+LABA) and placebo versus change in SGRQ total score at a study level for all study period**.

Using all treatment arms and all time points (n = 49), Figure 2 shows a moderate negative correlation between the mean change in trough FEV_1 _and change in SGRQ total score; greater increases in FEV_1 _were associated with greater reductions (i.e. improvements) in SGRQ. Zero change in FEV_1 _was associated with a significant reduction in SGRQ score of 2.5 (95% CI 1.8, 3.3). The additional reduction in SGRQ associated with a 100 mL increase in FEV_1 _was 1.6 (0.7, 2.5), making the total improvement in SGRQ 4.1 units. When excluding placebo arms, zero change in FEV_1 _was associated with a reduction in SGRQ total score of 4.1 (2.7, 5.6). However the association between change in FEV_1 _and additional change in SGRQ total score was no longer statistically significant; for a 100 mL increase in FEV_1 _the reduction in SGRQ was 0.4 (-1.1, 1.9).

Table 2 illustrates the increasing probability of reaching a clinically meaningful improvement in SGRQ with increasing levels of FEV_1 _improvement. For treatment arms where mean changes in FEV_1 _were ≥100 mL (using the largest ΔSGRQ values for studies with data for multiple time points) the probability of reaching a mean reduction in total SGRQ score of 4 units was 80%.

**Table 2:** Study level probability of reaching a clinically meaningful reduction in SGRQ total score according to the magnitude of improvement in FEV_1_

**Improvement in FEV**_**1 **_**(mL)**	Number of study arms achieving SGRQ reduction, n/N (%)	
	4 units	3 units
≥40	17/35 (49)	21/35 (60)
≥60	14/25 (56)	18/25 (72)
≥80	12/17 (71)	14/17 (82)
≥100	12/15 (80)	14/15 (93)
≥120	8/10 (80)	9/10 (90)

FEV_1_: forced expiratory volume in 1 second, SGRQ: St George's Respiratory Questionnaire

Random effects modelling found that a 100 mL increase in FEV_1 _was associated with an estimated reduction in SGRQ total score of 2.5 (1.9, 3.1). This equates to a clinically meaningful reduction of 4 units in SGRQ being associated with an estimated improvement in FEV_1 _of 160.6 (129.0, 211.6) mL. When this analysis was repeated excluding the placebo arms, a 100 mL increase in FEV_1 _led to an estimated change in SGRQ score of 1.02 (0.0, 2.5) although the association between FEV_1 _and SGRQ score was no longer significant.

### FEV_1 _change and SGRQ by study duration and SGRQ domains

As shown in Table 3, when data were analysed by time, change in trough FEV_1 _and change in SGRQ total score remained negatively correlated and the strength of the correlation increased with time for 3 (n = 16), 6 (n = 20) and 12 (n = 19) month time points. Reductions in SGRQ associated with zero change in FEV_1 _were 1.6 (95% CI -0.4, 3.6), 2.2 (1.1, 3.3) and 2.6 (1.8, 3.4), at 3, 6 and 12 months, respectively. Further reductions in SGRQ score associated with a 100 mL increase in FEV_1 _were 1.6 (-0.2, 3.5), 2.1 (1.3, 2.9) and 2.7 (1.5, 4.0) at 3, 6 and 12 months respectively.

**Table 3:** Correlations for mean change in FEV_1 _for all treatments (LAMA, LABA, LAMA + LABA) and placebo versus reduction in SGRQ scores at a study level, by study period and SGRQ domain

SGRQ	Study period	Data points, n	Correlation, r*	p value
Total score	All	49	-0.46	<0.001
	3 months	16	-0.44	0.08
	6 months	20	-0.61	0.004
	12 months	19	-0.74	<0.001
Symptoms	All	27	-0.34	0.08
Activity	All	27	-0.38	0.049
Impact	All	30	-0.50	0.004

*Pearson correlation coefficient

FEV1: forced expiratory volume in 1 second, LAMA: long-acting muscarinic antagonists, LABA: long-acting β2-agonists, SGRQ: St George's Respiratory Questionnaire

When data for all treatment and placebo arms, regardless of time, were stratified by SGRQ domains, there was a weak, non-significant negative correlation between change in trough FEV_1 _and change in SGRQ Symptoms score (Table 3). However there was a weak, but statistically significant negative correlation with change in SGRQ Activity score and a moderate and statistically significant negative correlation with change in SGRQ Impacts score.

### FEV_1 _change and other patient-reported outcomes

Table 4 presents the results for the relationship between change in FEV_1_, and TDI and exacerbations. Considering all treatment arms and 3, 6 and 12-month time points (n = 15), there was a moderate positive correlation between change in TDI and change in FEV_1_. The improvement in TDI associated with a 100 mL increase in FEV_1 _was 0.5 although this was below the 1 unit MCID for TDI [8]. When placebo arms were excluded from the analysis there was no evidence of an association between change in FEV_1 _and change in TDI score.

**Table 4:** Relationships between mean change in FEV_1 _and the outcomes Transition Dyspnea Index (TDI) and percentage of patients experiencing at least one exacerbation for all treatments (LAMA, LABA, LAMA + LABA) and placebo: correlation coefficients and model outputs

	Outcome	
	
	TDI total score	Percent of patients experiencing at least one exacerbation
Data points (n)	15	50
Correlation between change in outcome and change in FEV_1 _(r*)	0.56	-0.27
p value	0.02	0.049
Outcome change for 0 mL change in FEV_1 _(95% CI)**	0.7 (0.3, 1.2)	26.7 (21.7, 31.3)%
Additional change in outcome for 100 mL change in FEV_1 _(95% CI)**	0.5 (0.1, 0.9)	-6.0 (-0.04,-11.9)%

*Pearson correlation coefficient; **output from random effects regression modelling

FEV_1_: forced expiratory volume in 1 second, LAMA: long-acting muscarinic antagonists, LABA: long-acting β2-agonists

Increasing FEV_1 _was associated with a reduction in the proportion of patients experiencing at least one exacerbation, although the correlation was weak (Table 4). An increase of 100 mL in trough FEV_1 _was associated with an estimated 6.0% reduction in the proportion of patients experiencing at least one exacerbation. When placebo arms were excluded from the analysis (n = 33), the correlation was similar (r = -0.35; p = 0.046); zero change in FEV_1 _corresponded to an estimated 31.3% (95% CI 21.3, 41.3) of patients experiencing at least one exacerbation and a 100 mL increase in FEV_1 _was associated with an estimated 10.2% (0.2, 20.2) reduction in the numbers of patients experiencing an exacerbation.

## Discussion

Our study-level analysis demonstrated a relationship between improved lung function (as measured by FEV_1_) and improvements in health status (as measured by SGRQ) in adult patients with stable COPD who are treated with long-acting inhaled bronchodilators. Results of random-effects regression modelling indicated that a 100 mL increase in FEV_1 _was associated with a reduction in SGRQ total score of 2.5 units. This equates to a clinically meaningful reduction of 4 units in SGRQ being associated with an estimated improvement in FEV_1 _of 160.6 mL. These results were supported by correlation analyses which demonstrated a moderate negative correlation between change in total SGRQ score and change in trough FEV_1_, when all treatment arms were considered. When the placebo arms were excluded from the analyses the relationship was not significant, which may be due in part to the reduction in sample size, but principally because clustering of results for the placebo arms around zero for change in FEV_1 _and change in SGRQ increased the scatter in the data which allowed correlations to emerge. It should be emphasised that the principal objective of our review was to investigate the relationship between trough FEV_1 _and outcomes rather than test differential effects of treatment, so all use of treatment arms including placebo arms was appropriate. It is important to note that our analysis focussed on studies including long-acting bronchodilators. Relationships between FEV_1 _and outcomes may be different for anti-inflammatory treatments. Further, different results may have been obtained had we assessed the relationship between peak FEV_1 _and outcomes. However, we selected the trough measurement since it was the primary endpoint and therefore best documented outcome in most studies.

Despite the discrepancy in outcome measures required to demonstrate clinical effectiveness between the regulatory authorities and reimbursement agencies, such as the National Institute for Health and Clinical Excellence in the UK and the Institute for Quality and Efficiency in Health Care in Germany, few studies have investigated the relationship between change in lung function and change in patient-reported outcomes. We are aware of no other analysis addressing this issue at a study level. However, our data are consistent with the results of patient-level analyses [5,48], although in these studies the strength of the relationship between change in SGRQ and FEV_1 _was too weak to allow health status gains to be inferred from spirometric changes [48]. This is not a limitation, but rather reflects how different individuals with the same physiological limitations may experience differing effects on their health status.

Our study indicated that the correlation between change in trough FEV_1 _and change in SGRQ total score appears to strengthen with increasing study duration from 3 to 6 to 12 months. Over an intermediate and longer term period, the impact of an improvement in lung function may have a greater effect on patient well-being, although in our analysis, the limited data reported in the included studies did not allow us to assess whether changes in FEV_1 _at 3 months correlated with longer term changes in outcomes. There was also a trend to increasing mean change in SGRQ, across all study arms, with longer study duration. When data were analysed by SGRQ domain, the association between change in FEV_1 _and change in SGRQ scores was still present for the Activity and Impacts domains. A weak correlation between SGRQ Symptoms domain and FEV_1 _has been reported ever since the first validation of this instrument [3].

Another important issue to be addressed is the "meaning" of the 100 mL increase in FEV_1 _associated with a reduction in SGRQ total score of 2.5 units, and an estimated improvement in FEV_1 _of 160 mL in relation to a clinically meaningful reduction of 4 units in SGRQ. There is no universally accepted approach for determining the clinical important difference of a measurement. As a measure, SGRQ reflects aspects of COPD beyond lung function alone [48]. In our analysis, the corresponding increase in health status in treatment arms with larger improvement in FEV_1 _enhances the ability to interpret lung function changes at a study level, but not at a patient level. Depending on the intervention under study, FEV_1 _may offer the perspective of an intermediate end point in assessing likely treatment effectiveness. However, treatment effectiveness cannot be based exclusively on spirometry, requiring assessment of other relevant clinical parameters such as patient-reported health status.

It is interesting to note that a zero change in FEV_1 _still resulted in a reduction in SGRQ score of 2.5. This effect has been noted in many clinical trials in COPD and appears to relate to a 'Hawthorne effect', whereby patients receive better care by participating in the trial [49]. It could relate to a number of different factors, including improved compliance with treatments which may not all have bronchodilator effects.

There was also some evidence of a positive relationship between change in FEV_1 _and other outcomes, i.e., improvements in TDI score and reduction in the proportion of patients experiencing at least one exacerbation. These associations were weaker than those observed with SGRQ. However, correlation data for TDI versus trough FEV_1 _were limited by the relatively small number of studies (n = 8) reporting both outcome measures. For data on exacerbations, longer study durations would have been required to fully assess the apparent negative correlation with change in FEV_1_.

Our review has limitations. We did not explicitly seek primary studies assessing the correlation between outcome measures and the restriction of our search strategy to RCTs in order to enhance the quality of the analysis means that observational studies of this type would not have been identified. In addition, the objectives of included studies differed from those of the review: included studies were generally designed to measure the effects of treatment upon COPD outcomes, whereas we were interested in the relationships between outcome measures. Included studies tended to present full results for their primary outcome measure only, with reporting of additional outcomes being poor and measures of variance were often absent. Thus, standard deviations had to be imputed for a high proportion of the data sets included in our analyses. In addition, many studies did not report numerical data and values were estimated from graphs, although such approaches are consistent with established systematic review methodology.

Although our review did not address treatment effect sizes, our objectives did include an assessment of the relationships between treatment effects upon treatment effect sizes (data addressing this objective were sparse and not included in this article). For this reason only RCTs of long acting bronchodilators which included a placebo arm or which compared different classes of bronchodilator were compared.

Finally, the correlation analyses used to assess the relationships between patient-reported outcomes and FEV_1 _where data were insufficient to support regression modelling, combined treatment arms from different studies. Thus the data were essentially treated as observational cohorts and the strengths of the RCT design were lost. Combining the data in this way does not take account of differences between studies, such as treatment and dose, and participant baseline characteristics, which may affect estimates of correlation. In theory, this limitation can be overcome using random effects regression modelling. However, even where such modelling was possible, the number of explanatory variables which could be included was constrained by both the reporting of these variables in the primary studies and the size of the data set; both poor reporting and small data sets were factors in this review.

The results of this review give important new insight into the relationship between FEV_1_, a key primary outcome required by regulatory authorities for COPD clinical trials, and patient-reported outcomes such as health status, dyspnoea and exacerbations, which are of greater interest to clinicians, patients and reimbursement agencies. Our analyses have been limited by the size and quality of the available data set and are encouraging, but should be considered hypothesis generating and warrant further investigation.

This study-level analysis indicated that improvement in trough FEV_1 _with inhaled bronchodilators may be associated with improvement in health status and may also be associated with improvements in other patient-reported outcomes. Although the strength of the association was modest, improvements in both FEV_1 _and SGRQ, relative to changes likely to be clinically relevant, were of similar magnitude. FEV_1 _may offer the perspective of an intermediate endpoint in assessing treatment effectiveness at a study level.

## Competing interests

AC and GCN are employees of Novartis. MW and GW have no competing interests related to the content of this paper. JB has received fees for speaking at conferences and for serving as an expert on advisory boards for AstraZeneca, BI, GSK, Novartis, Nycomed and Pfizer. JB's MUHC Research Institute received research grants for investigator-initiated researches and unrestricted educational grants from AstraZeneca, BI, GSK, Novartis and Pfizer. PJ has received advisory board and consulting fees from Novartis, GSK, AZ, Boehringer, Roche, Almirall and Spiration. He has received speaker's fees from GSK.

## Authors' contributions

MW developed the design, concept of the study and analysis, and carried out the systematic review. JB participated in the design and analysis planning and advised on the interpretation of the study. PWJ participated in the design and advised on the interpretation of the study. AC conceived of the study, participated in its design and analysis planning and contributed to its interpretation. GCN conceived of the study, participated in its design and analysis planning and contributed to its interpretation. GW developed the design and concept of the study, carried out the systematic review and performed the statistical analysis. All authors had full access to the data and were involved in drafting the manuscript. All authors read and approved the final manuscript.

## Supplementary Material

Additional file 1 **Search strategy for the MEDLINE database**. Click here for file

Additional file 2 **Quality assessment of studies selected for inclusion in the systematic review**. Click here for file
