# Supplementary material for: Relationship between FEV1 change and patient-reported outcomes in randomised trials of inhaled bronchodilators for stable COPD: a systematic review
Source: Respir Res. 2011 Apr 8;12(1):40. doi: 10.1186/1465-9921-12-40 (PMC3090353; doi:10.1186/1465-9921-12-40)
Supplement: Additional file 1 — Search strategy for the MEDLINE database. [file 1465-9921-12-40-S1.DOCX]

**Table S1:** Search strategy – MEDLINE (1950 – 11^th^ June 2009)

| # | Search term | Results |
| --- | --- | --- |
| 1 | copd.mp. or exp Pulmonary Disease, Chronic Obstructive/ | 19404 |
| 2 | coad.mp. | 182 |
| 3 | (chronic adj3 bronchitis).mp. [mp=title, original title, abstract, name of substance word, subject heading word] | 9298 |
| 4 | Emphysema.mp. | 23599 |
| 5 | airflow obstruction.mp. | 2511 |
| 6 | wheeze$.mp. | 2831 |
| 7 | hyperinflation.mp. | 1699 |
| 8 | exp Bronchodilator Agents/ | 192395 |
| 9 | exp Adrenergic beta-Agonists/ | 96036 |
| 10 | exp Adrenal Cortex Hormones/ | 291146 * |
| 11 | exp Cholinergic Antagonists/ | 67514 |
| 12 | exp Muscarinic Antagonists/ | 45368 |
| 13 | exp clinical trials/ | 577437 |
| 14 | exp research design/ | 254139 |
| 15 | exp treatment outcome/ | 393968 |
| 16 | exp double-blind method/ | 101886 |
| 17 | exp single-blind method/ | 12985 |
| 18 | ((single or double or triple) adj3 blind$3).ti,ab,hw. | 139697 |
| 19 | random$.ti,ab,hw. | 611342 |
| 20 | controlled clinical trial.pt. | 79488 |
| 21 | clinical trial.pt. | 453627 |
| 22 | (clinical adj trial$1).ti,ab,hw. | 634850 |
| 23 | exp epidemiological research design/ | 597617 |
| 24 | (control$3 adj trial$1).ti,ab,hw. | 354244 |
| 25 | randomi#ed controlled trial.pt. | 273354 |
| 26 | comparative study/ | 1439045 |
| 27 | placebo$.ti,ab,hw. | 133075 |
| 28 | or/1-7 | 54113 |
| 29 | or/8-12 | 520445 |
| 30 | or/13-27 | 2819844 |
| 31 | and/28-30 | 2557 |
| * Studies of inhaled corticosteroids were excluded from the review after initial searches had been completed. | | |
