# Supplementary material for: Relationship between FEV1 change and patient-reported outcomes in randomised trials of inhaled bronchodilators for stable COPD: a systematic review
Source: Respir Res. 2011 Apr 8;12(1):40. doi: 10.1186/1465-9921-12-40 (PMC3090353; doi:10.1186/1465-9921-12-40)
Supplement: Additional file 2 — Quality assessment of studies selected for inclusion in the systematic review. [file 1465-9921-12-40-S2.DOCX]

**Table S2.** Quality assessment of studies selected for inclusion in the systematic review

| Criteria | Aaron 2007 [11] | Baumgartner 2007 [12] | Beeh 2006 [32] | Boyd 1997 [33] | Briggs 2005 [34] | Brusasco 2003 [13] |
| --- | --- | --- | --- | --- | --- | --- |
| 1. Was the allocation sequence adequately generated? | Yes | Not reported | Not reported | Not reported | Not reported | Not reported |
| 2. Was allocation adequately concealed? | Yes | Not reported | Not reported | Not reported | Not reported | Not reported |
| 3. Was knowledge of the allocated interventions adequately prevented during the study? | Yes | Yes | Yes | Yes | Yes | Yes |
| 4. Were baseline characteristics similar across groups? | Yes | Yes | Yes | Yes | Yes | Yes |
| 5. Were incomplete outcome data adequately addressed? | Yes | Yes | No | No | No | No |
| 6. Are reports of the study free of suggestion of selective outcome reporting? | Yes | Yes | Yes | No | Yes | No |
| 7. Was the study apparently free of other problems that could put it at a high risk of bias? | Yes | Yes | Yes | Yes | Yes | Not reported |

| Criteria | Calverley 2003 [14] | Calverley 2007 [15] | Campbell 2005 [16] | Casaburi 2000 [35] | Casaburi 2002 [17] | Chan 2007 [18] |
| --- | --- | --- | --- | --- | --- | --- |
| 1. Was the allocation sequence adequately generated? | Yes | Yes | Yes | No | No | Not reported |
| 2. Was allocation adequately concealed? | Yes | Yes | Yes | No | No | Not reported |
| 3. Was knowledge of the allocated interventions adequately prevented during the study? | Yes | Yes | Yes | Yes | Yes | Yes |
| 4. Were baseline characteristics similar across groups? | Yes | Yes | Yes | Yes | Yes | Yes |
| 5. Were incomplete outcome data adequately addressed? | Yes | Yes | No | No | No | Yes |
| 6. Are reports of the study free of suggestion of selective outcome reporting? | Yes | No | No | No | No | Yes |
| 7. Was the study apparently free of other problems that could put it at a high risk of bias? | Not reported | Yes | Yes | Yes | Yes | Yes |

| Criteria | Chapman 2002 [19] | Covelli 2005 [36] | Dahl 2001 [20] | Donohue 2002 [21] | Donohue 2008 [37] | Dusser 2006 [38] |
| --- | --- | --- | --- | --- | --- | --- |
| 1. Was the allocation sequence adequately generated? | Not reported | Not reported | Not reported | Not reported | Not reported | Not reported |
| 2. Was allocation adequately concealed? | Not reported | Not reported | Not reported | Not reported | Not reported | Not reported |
| 3. Was knowledge of the allocated interventions adequately prevented during the study? | Yes | Yes | Yes | Yes | No | Yes |
| 4. Were baseline characteristics similar across groups? | Yes | Yes | Yes | Yes | Yes | Yes |
| 5. Were incomplete outcome data adequately addressed? | No | No | No | No | Yes | No |
| 6. Are reports of the study free of suggestion of selective outcome reporting? | No | No | No | No | Yes | Yes |
| 7. Was the study apparently free of other problems that could put it at a high risk of bias? | Yes | No | Not reported | Not reported | Yes | Yes |

| Criteria | Freeman 2007 [39] | Gross 2008 [22] | Johansson 2008 [40] | Jones 1997 [23] | Mahler 1999 [41] | Moita 2008 [42] |
| --- | --- | --- | --- | --- | --- | --- |
| 1. Was the allocation sequence adequately generated? | Not reported | Not reported | Not reported | Not reported | Not reported | Not reported |
| 2. Was allocation adequately concealed? | Not reported | Not reported | Not reported | Not reported | Not reported | Not reported |
| 3. Was knowledge of the allocated interventions adequately prevented during the study? | Yes | Yes | Yes | Yes | Yes | Yes |
| 4. Were baseline characteristics similar across groups? | Yes | Yes | Yes | Yes | Yes | Yes |
| 5. Were incomplete outcome data adequately addressed? | Yes | Yes | No | No | No | Yes |
| 6. Are reports of the study free of suggestion of selective outcome reporting? | Yes | No | No | Yes | No | No |
| 7. Was the study apparently free of other problems that could put it at a high risk of bias? | Yes | No | No | Not reported | Not reported | No |

| Criteria | Niewoehner 2005 [43] | Rennard 2009 [24] | Rossi 2002 [25] | Sepracor inc.  NCT00250679 2009 [26] | Stahl 2001 [5] | Stockley 2006 [27] |
| --- | --- | --- | --- | --- | --- | --- |
| 1. Was the allocation sequence adequately generated? | Yes | Not reported | Not reported | Not reported | Not reported | Yes |
| 2. Was allocation adequately concealed? | Yes | Not reported | Not reported | Not reported | Not reported | Not reported |
| 3. Was knowledge of the allocated interventions adequately prevented during the study? | Yes | Yes | Yes | Yes | Yes | Yes |
| 4. Were baseline characteristics similar across groups? | Yes | Yes | Yes | Yes | Yes | Yes |
| 5. Were incomplete outcome data adequately addressed? | Yes | Yes | Yes | Yes | No | Yes |
| 6. Are reports of the study free of suggestion of selective outcome reporting? | Yes | Yes | No | Yes | No | No |
| 7. Was the study apparently free of other problems that could put it at a high risk of bias? | Yes | Yes | Yes | Yes | No | Yes |

| Criteria | Tashkin 2008a [28] | Tashkin 2008b [29] | Tashkin 2009 [30] | Tonnel 2008 [31] | van Noord 2000 [44] | Vogelmeier 2008 [45] |
| --- | --- | --- | --- | --- | --- | --- |
| 1. Was the allocation sequence adequately generated? | Yes | Yes | Yes | Yes | Not reported | Not reported |
| 2. Was allocation adequately concealed? | Not reported | Not reported | Not reported | Not reported | Not reported | Not reported |
| 3. Was knowledge of the allocated interventions adequately prevented during the study? | Yes | Yes | Yes | Yes | Yes | Yes |
| 4. Were baseline characteristics similar across groups? | Yes | Yes | No | Yes | Yes | Yes |
| 5. Were incomplete outcome data adequately addressed? | Yes | Yes | Yes | Yes | Yes | Yes |
| 6. Are reports of the study free of suggestion of selective outcome reporting? | Yes | No | No | Yes | No | No |
| 7. Was the study apparently free of other problems that could put it at a high risk of bias? | Yes | Yes | Yes | Yes | Yes | Yes |
